# 48. Primary bone marrow oedema syndrome

**DOI:** 10.1093/rap/rky034.011

**Published:** 2018-09-20

**Authors:** Shawki El-Ghazali, Kuljeet Bhamra, Ajmal Khan, Mitchell Burden, Ishita Patel

**Affiliations:** Rheumatology, Wexham Park Hospital, Slough, UNITED KINGDOM

**Introduction:** Bone marrow oedema syndrome is an uncommon condition resulting in bone/joint pain often over a chronic period. Diagnosis is typically made based on MRI findings identified as in our case. Symptoms can be transient or prolonged, and often diagnosis can be missed. We present a case of this atypical condition of a patient seen within our department.

**Case description:** A 59 year old female of Somalian origin attended hospital with an eight week history of left foot pain and swelling. She had been experiencing similar symptoms of the right foot, however this had resolved a few weeks prior to presentation. Prior to attendance, she had been using NSAID therapy with limited benefit. She denied any trauma or any specific event resulting in symptoms. She had been experiencing similar episodes of foot pain and swelling in the past going back 10 years. Episodes were sporadic and would self-resolve over weeks to months and prior to current presentation, no formal diagnosis had been attributed to these events. Past medical history included hypothyroidism, L4/L5 canal stenosis and depression. On examination she was noted to have generalised oedema of the left foot - primarily of the dorsum and ankle. She exhibited allodynia and shiny skin of the affected region. No evidence of skin cellulitis was identified and no other part of the body appeared to be affected. Systemic examination was otherwise normal, with no other joint involvement. She denied history or other symptoms suggestive of typical inflammatory joint or connective tissue disease. Initial investigations demonstrated raised CRP of 31 and ESR 88. Urate was 460 umol/L. IgG was raised at 28.2 g/L (6-16 g/L). Vitamin D was low at < 20, for which replacement therapy was initiated. ALP was mildly raised at 161 IU/L (35-104 IU/L), however remaining LFTs were normal, as was calcium levels. Blood tests were otherwise unremarkable with normal FBC, renal function, serum ACE, rheumatoid factor and anti-CCP levels. dsDNA, ANA, ENA and complement levels were also within normal range. HIV screen was negative. X-ray of the left foot demonstrated mild osteopenia but otherwise did not report any focal bony lesion or radiological evidence of osteomyelitis. CT chest/abdomen/pelvis was arranged to review possible underlying cause to account for the patient's symptoms. This did not report any focal lesion, mediastinal lymphadenopathy or any significant finding. MRI of the left foot and ankle was performed and this reported to show diffuse marrow oedema of the mid and hindfoot. No erosions or synovitis was identified. No obvious cause was identified to explain described MRI findings. Review of the case within radiology MDT presented a possible diagnosis of primary bone marrow oedema syndrome. Over time, symptoms slowly improved with conservative measures including analgesia, elevation and use of a walking boot to alleviate weight-bearing. Possible pharmacological treatments have been suggested within the literature, including bisphosphonate therapy and during admission, she received a single dose of IV pamidronate (60mg). Follow up has been arranged for monitoring within the community. Review shortly after discharge demonstrated continued gradual improvement of symptoms.

**Discussion:** Bone marrow oedema syndrome is a clinico-radiological diagnosis made in the context of transient subacute or chronic joint pain in the presence of typical MRI findings. MRI changes consist of high signal of the bone marrow in T2-weighted images, intermediate signal in T1-weighted, and hyperintensity of the bone marrow in contrast enhanced/STIR imaging. Bone marrow oedema in general can occur as a secondary process to other pathology including trauma, osteoarthritis, sickle cell, infection and neoplasm. In cases where no obvious underlying cause can be identified, then findings consistent with radiological changes are termed primary bone marrow oedema syndrome. Exact aetiology of this particular form has not been fully identified. Possible suggested causes include local ischaemia resulting in increased bone turnover which in turn causes localised marrow oedema. Cases are typically seen of the lower limb, usually of the hip and less frequently of the knees and ankles/feet. Symptom duration can vary from weeks to months. Suggested management is primarily conservative, with avoidance of weight bearing and appropriate analgesia. Evidence for pharmacological treatment is limited, however reported benefit of bisphosphonates, iloprost, calcium channel blockers and glucocorticosteroids have been described within the literature. Surgical core decompression has additionally been suggested in patients with severe and difficultly controlled pain, although in view of the typical self-resolution of symptoms, this can be seen as a last resort. Regarding our case, particular features stand out from more standard presentations of this condition. As mentioned, hips are the most common site of pathology, with decreasing incidence at the distal extremity of the lower limb. In our case, only the foot and ankle region was affected, with both feet being affected at different points of time. Recurrence of episodes have been described in the literature, and can affect different anatomical sites. This was the case for our patient, but specifically affected the distal limb on each occasion rather than more common proximal sites. Episodes had been relatively frequent also in our patient, with multiple episodes over the preceding 1ten years - which also appears to be an atypical phenomenon. Due to the location of symptoms at the patient's feet, her recurrent episodes had been essentially misdiagnosed as prolonged gout flares, thus leading to delayed diagnosis. Unfortunately, based on the literature, this is a more common experience in patients with foot involvement due to its lower frequency and non-specific nature. As such, patients are at risk of persistent bone pain symptoms which can affect function and subsequent quality of life.

**Key Learning Points:** Bone marrow oedema syndrome is a rare condition that can often be missed. Symptoms can be transient in nature with patients not presenting, or being misdiagnosed with alternative conditions. In our described case, the patient's episode was prolonged thus allowing appropriate investigation. Cases are generally managed conservatively, however gradual resolution can take weeks to months to occur. Literature is limited in regarding medical therapy, however use of bisphosphonates has been described and was utilised in this case. Our patient is undergoing continued follow up within the community under the rheumatology team to monitor her gradual recovery.

**Disclosure: S. El-Ghazali:** None. **K. Bhamra:** None. **A. Khan:** None. **M. Burden:** None. **I. Patel:** None.

